# Modelling stromal compartments to recapitulate the ameloblastoma tumour microenvironment

**DOI:** 10.1016/j.mbplus.2022.100125

**Published:** 2022-11-21

**Authors:** Deniz Bakkalci, Amir Zaki Abdullah Zubir, Syed Ali Khurram, Judith Pape, Kristiina Heikinheimo, Stefano Fedele, Umber Cheema

**Affiliations:** a UCL Centre for 3D Models of Health and Disease, Division of Surgery and Interventional Science, University College London, Charles Bell House, 43-45 Foley Street, W1W 7TS London, UK; b Unit of Oral and Maxillofacial Pathology, School of Clinical Dentistry, University of Sheffield, 19 Claremont Crescent, S10 2TA Sheffield, UK; c Department of Oral and Maxillofacial Surgery, Institute of Dentistry, University of Turku and Turku University Hospital, Turku, Finland; d Eastman Dental Institute, University College London, London, UK

**Keywords:** AM, Ameloblastoma, α-SMA, alpha-smooth muscle actin, BPE, Bovine pituitary extract, BSA, Bovine serum albumin, CCN, Cellular communication network factor 2, dl-CGH, Double-layered collagen gel hemisphere, DMEM, Dulbecco’s modified Eagle medium, ECM, Extracellular matrix, FBS, Foetal bovine serum, HFF-2, Human fibroblasts (HFF-2), HGF, Primary gingival fibroblasts, hOB, Human osteoblasts, IF, Immunofluorescence, H&E, Haematoxylin and eosin, IHC, Immunohistochemistry, MEM, Minimal Essential Medium, MIQE, Minimum Information for Publication of Quantitative Real-Time PCR Experiments, MMPs, Matrix metalloproteinases, M, Molar, Min, Minutes, N.A, Neutralising agent, MRC5, Human lung fibroblasts, PTHLH, Parathyroid Hormone Like Hormone, RANK, The tumour necrosis factor (TNF) superfamily members receptor activator of nuclear factor kappa-B receptor, RANKL, The tumour necrosis factor (TNF) superfamily members receptor activator of nuclear factor kappa-B ligand *(TNFSF11)*, RT, Room temperature, SD, Standard deviation, SEM, Standard error mean, SPARC, anti-osteonectin, TGF-β, Transforming growth factor, TME, Tumour microenvironment, TNF, Tumour necrosis factor, Fibroblasts, 3D models, IHC, RANKL, Tumour microenvironment

## Abstract

Tumour development and progression is dependent upon tumour cell interaction with the tissue stroma. Bioengineering the tumour-stroma microenvironment (TME) into 3D biomimetic models is crucial to gain insight into tumour cell development and progression pathways and identify therapeutic targets. Ameloblastoma is a benign but locally aggressive epithelial odontogenic neoplasm that mainly occurs in the jawbone and can cause significant morbidity and sometimes death. The molecular mechanisms for ameloblastoma progression are poorly understood. A spatial model recapitulating the tumour and stroma was engineered to show that without a relevant stromal population, tumour invasion is quantitatively decreased. Where a relevant stroma was engineered in dense collagen populated by gingival fibroblasts, enhanced receptor activator of nuclear factor kappa-B ligand (RANKL) expression was observed and histopathological properties, including ameloblastoma tumour islands, developed and were quantified. Using human osteoblasts (bone stroma) further enhanced the biomimicry of ameloblastoma histopathological phenotypes. This work demonstrates the importance of the two key stromal populations, osteoblasts, and gingival fibroblasts, for accurate 3D biomimetic ameloblastoma modelling.

## Introduction

Tumour development and progression rely on its interaction with the surrounding stroma. The extracellular matrix (ECM) including stromal cells such as fibroblasts and adipose tissue, immune cells and the bone form the tumour microenvironment (TME) [1]. The TME helps to direct and orchestrate multiple facets of tumour development, including angiogenesis, invasion, and metastasis. The TME consists of stromal cells within a specific physical niche, which is unique to each tumour. Recapitulating the tumour microenvironment begins with determining favourable conditions including a 3D environment with the appropriate ECM composition, architecture, and the relevant stromal cell populations [2]. Until now *in vitro* ameloblastoma models have helped to decipher elements of disease pathophysiology, however more complex models are required to test therapeutic strategies. Therefore, this work aimed to understand the impact of relevant and biomimetic stromal cells on ameloblastoma tumour mass in a compartmentalised 3D tumouroid model particularly with regards to tumour invasion.

Studying rare diseases can be challenging since patient tissue and cells are limited and the understanding of disease mechanism is limited. Ameloblastoma (AM) is a rare odontogenic tumour with benign but locally aggressive behaviour [3]. The most common treatment is the wide resection of jaw (similar to a malignant neoplasm). However, the resection leaves patients with postoperative facial deformities as well as problems in eating and speaking [4], [5].

The two most common histopathological types of AM are follicular and plexiform. The follicular type resembles the epithelial components of the enamel organ, whereas the plexiform type presents as anastomosing cords and strands of ameloblastomatous epithelium [6]. The stroma of ameloblastoma is composed of mature fibrous tissue and the neoplasm typically grows slowly but progressively to the surrounding bone. Ameloblastoma forms within a rich and complex stroma where ingrowth and intrusion of tumour cells into the surrounding tissues is observed. To date, the focus on studying ameloblastoma has been towards investigating how it induces bone resorption [7], [8]. The tumour necrosis factor (TNF) superfamily members receptor activator of nuclear factor kappa-B receptor (RANK) and its ligand (RANKL) have a crucial role in differentiation and activation of the bone resorbing cells, osteoclasts. Current understanding specifies that ameloblastoma cells cause bone resorption by initiating osteoclastogenesis via the RANK/RANKL/osteoprotegerin (OPG) pathway and the release of matrix metalloproteinases (MMPs) [9]. However, the precise molecular mechanisms behind how ameloblastoma impacts bone homeostasis are unknown. There is some evidence to suggest that ameloblastoma cells can disrupt bone homeostasis by interfering with both bone formation and bone resorption [10].

**Fig. 1:**
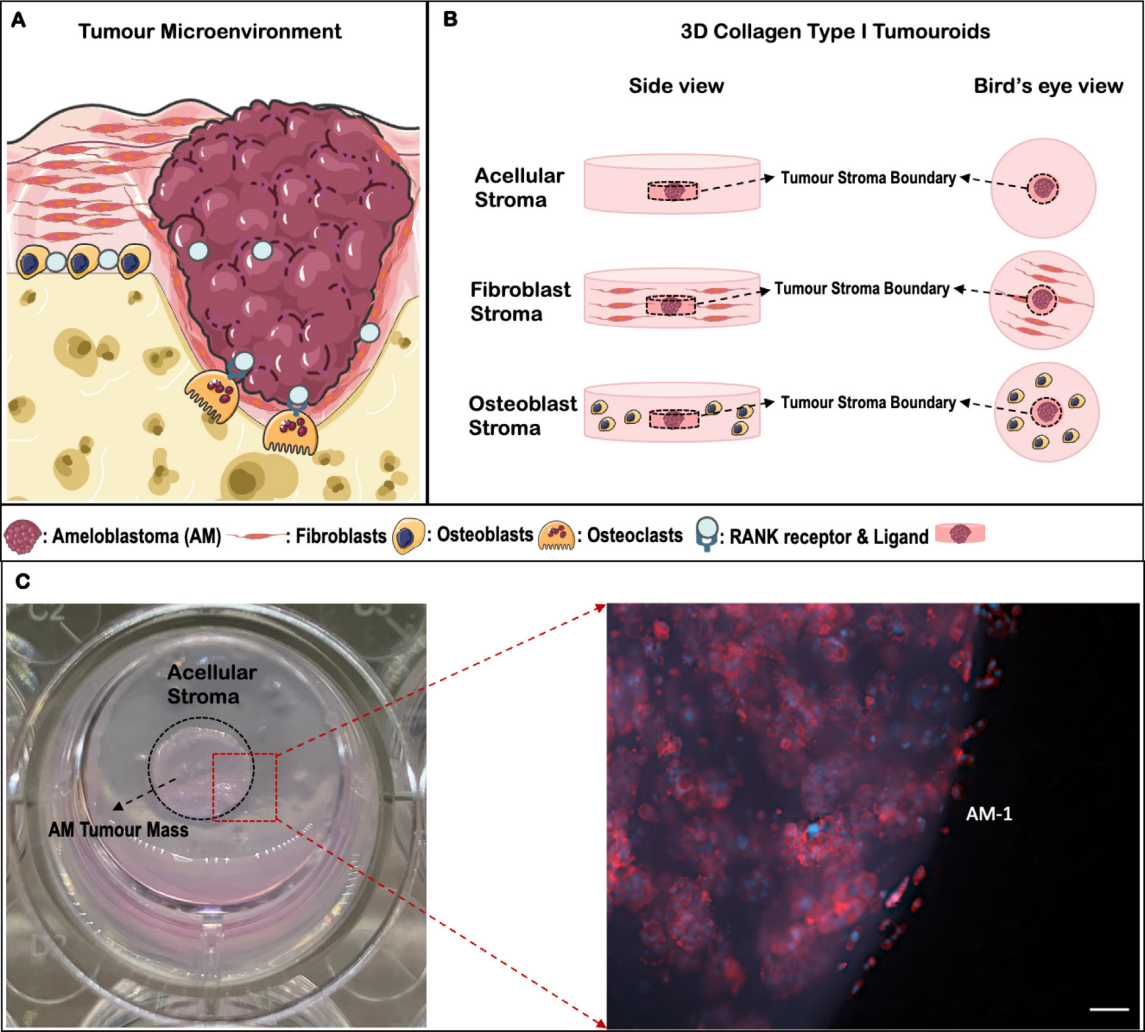
Schematic of ameloblastoma tumour microenvironment (A) and 3D Collagen Tumouroids (B). The figure was created using Servier Medical Art. (C) The photo of the 3D tumouroid model representing Bird’s eye view of AM tumour mass, acellular stroma, and the region of interest. The tumour-stroma boundary imaged following Immunofluorescence staining and the IF image is AM-1 tumouroid at day 7, red = Phalloidin, blue = DAPI, scale bar = 100 µm. (For interpretation of the references to colour in this figure legend, the reader is referred to the web version of this article.)

A number of tissue models have been proposed to study ameloblastoma, however it is still important to develop novel models to better represent ameloblastoma tumour microenvironment. There is increasing evidence of ameloblastoma tumour-stroma interactions. Furthermore, bone cells and fibroblasts comprise a large proportion of the ameloblastoma TME (Fig. 1A). The role of resident fibroblasts has been highlighted by Takebe *et al*, who showed ameloblastoma induced fibrosis and osteoclastogenesis by studying Cellular Communication Network Factor 2 (CCN2) and Transforming Growth Factor (TGF-β) pathways [11]. In a different study, co-culture of human fibroblasts (HFF-2) and ameloblastoma cell lines (AM-1 and AM-3) in a double-layered collagen gel hemisphere (dl-CGH) showed that the fibroblasts promoted collective ameloblastoma invasion similar to the follicular variant of ameloblastoma [12]. It is well established that tumour cells are stimulated by the fibroblasts. The study by [13] showed a positive correlation between alpha-smooth muscle actin (α-SMA) and stem cell markers and suggested that oral cancer stem cells induce tumourigenesis by activating stromal fibroblasts [13]. In the ameloblastoma TME, fibroblasts usually surround ameloblastoma cells [11]. Myofibroblasts which are smooth muscle-like fibroblast with contractile apparatus have been detected in ameloblastoma patient samples using alpha-smooth muscle actin (α-SMA) immunohistochemistry (IHC). The myofibroblasts have a different secretory phenotype to resting fibroblasts with an ability to induce development to induce development of neoplastic epithelial lesions [14].

3D model research has primarily focused on understanding how to the mimic the TME. A previously established 3D ameloblastoma tumouroid model incorporated an active bone forming stroma and investigated ameloblastoma and its association with its surrounding stroma [10]. This current study furthers our understanding of how ameloblastoma stromal cells, both gingival fibroblasts and osteoblasts, enable biomimicry of ameloblastoma phenotypes in 3D tumouroid models.

## Methods

### Cell culture

All cultures were maintained in 37 °C, 5 % CO_2_, and 21 % O_2_. Prof Harada kindly provided AM-1 cells (immortalised from plexiform ameloblastoma) [15]. AM-3 cells (immortalised from follicular ameloblastoma) were a gift from Prof Kishida and colleagues [12]. AM-1 and AM-3 cells were cultured in keratinocyte serum free medium 1X (KSFM) supplemented with KSFM supplements bovine pituitary extract (BPE) and epidermal growth factor (EGF), human recombinant and defined KSFM with DKSFM supplement respectively. Primary gingival fibroblasts, Human, Adult (HGF) (PCS-201-018™) and MRC-5, lung fibroblasts (CCL-171^TM^) were obtained from ATCC and cultured in Dulbecco’s modified Eagle medium (DMEM). The human osteoblasts (hOB) were obtained from Promocell® (Heidelberg, Germany) and cultured in osteoblast’s growth medium with supplement mix. All media types were supplemented with 10 % Foetal bovine serum (FBS), 100 units/mL penicillin, and 100 μg/mL streptomycin (all from Gibco^TM^ through Thermo Fisher Scientific, Loughborough, UK).

### 3D model fabrication

All 3D models were fabricated from monomeric type I collagen (First Link, Birmingham, UK). RAFT^TM^ protocol was followed throughout the fabrication (Lonza, Slough, UK). The collagen/cell mix was prepared from 10X Minimal Essential Medium (MEM) (Sigma-Aldrich, Dorset, UK), collagen, neutralising agent (N.A) and cells. The volumes of the mix were 80 % collagen, 10X MEM, 6 % N.A. and 4 % cells and it was kept on ice. The N.A. was made of 17 % 10 Molar (M) NaOH (Sigma-Aldrich, Dorset, UK) and 83 % 10 M HEPES buffer (Gibco^TM^ through Thermo Fisher Scientific, Loughborough).

The tumour mass was prepared by culturing 240 μl of cell/collagen mix with either 5x10^4^ AM-1 cells or AM-3 cells per well into 96-well plates (Corning® Costar®, Sigma-Aldrich, Dorset, UK). The gel mix was crosslinked at 37 °C for 15 min followed by 15 min plastic compression using the RAFT^TM^ absorbers at room temperature (Lonza, Slough, UK). The complex tumouroids were prepared by embedding a tumour mass within the stromal compartment composed of either HGFs (7 × 10^4^) or hOBs (7 × 10^4^) (Fig. 1 B). Initially, 650 μl of the stromal gel mix was cast on 24-well plate (Corning® Costar ®, Sigma-Aldrich, Dorset, UK) followed by placing the tumour mass was placed in the middle. A second layer of 650 μl of the stromal gel mix was applied on top (Fig. 1B).

Tumouroids were crosslinked for 15 min at 37 °C, and plastic compressed using 24-well RAFT^TM^ absorbers (Lonza, Slough, UK) for 15 min at room temperature. 2 ml of media was added to the gels with 50 % media change in every 48 h. For the mineralisation of hOB, mineralisation medium with the supplement mix was used in 3D hOB cultures (Promocell, Heidelberg, Germany). Three experimental repeats per condition were prepared. The cultures were kept for 14 days.

### Imaging and measuring invasion

**Fig. 2:**
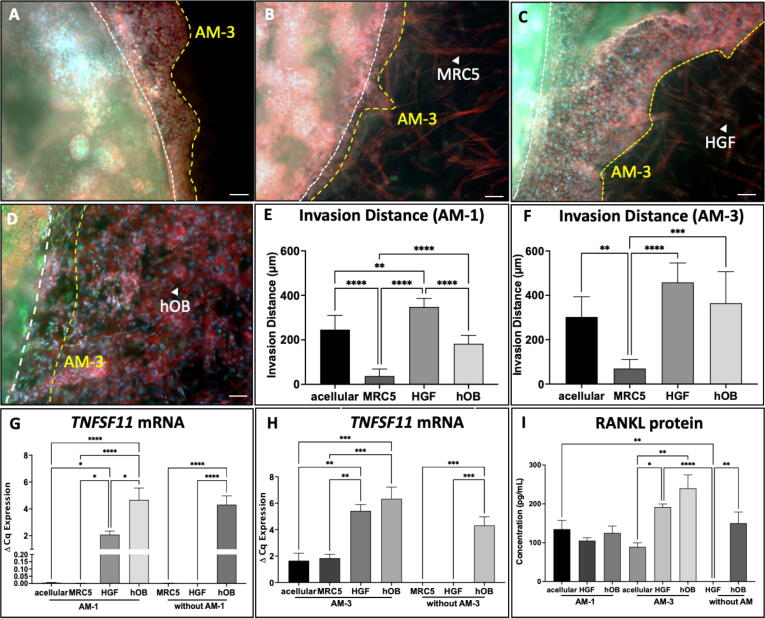
Adding native stromal cells promotes ameloblastoma invasion compared to acellular. Invasion of AM-1 cells from the tumour mass to (A) acellular (A), (B) MRC5, (C) and to (D) hOB stroma at day 14, red = Phalloidin, green = RANKL, blue = DAPI, scale bar = 100 µm. White lines describe tumour mass and stroma boundary and yellow lines represent invasion of tumour cells to the surrounding stroma. (E&F) Invasion distance of AM-1 and AM-3 cells to HFG and MRC5 stroma at day 14. mRNA expression of (G&H) TNFSF11 (RANKL) in AM-1 and AM-3 tumoroids with acellular stroma, MRC5, HGF, and hOB stroma and controls of HGF and hOB only tumouroids at day 14. (I) RANKL protein concentration in AM-1 and AM-3 tumouroids at day 14. All values were measured as n = 3. One-Way ANOVA, Dunnet’s Post Hoc; p-values 0.05 < *, 0.005 < **, 0.0005 < *** and 0.00005 < ****. (For interpretation of the references to colour in this figure legend, the reader is referred to the web version of this article.)

The 3D samples were imaged using the Zeiss AxioObserver with Apotone.2 instrument and software (Zeiss, Oberkochen, Germany) as described previously [16]. Spheroids were defined as clustering of cells as spheroids, where invasion was seen as the outgrowth of tumour cells from the tumour mass boundary towards the surrounding stroma (Fig. 1C). In Fig. 2 A-D, the white lines represent tumour mass boundary, where the yellow lines demonstrate the invasion of tumour cells. Invasion distance was the distance of tumour cells that had invaded to the stroma from the main tumour mass. ImageJ (NIH, USA) was used for image analysis.

### RNA extraction, cDNA synthesis, quantitative polymerase chain reaction (qPCR)

TRI Reagent® was used for RNA extraction steps beginning with phase separation followed by the chloroform method [17]. Three replicates were extracted for each condition and each replicate was created from a pool of three 3D samples. RNA quality and quantity were assessed by using Nano-Drop^TM^ Transcription of RNA into cDNA was performed on T100^TM^ Thermal Cycler (Bio-Rad, Watford, UK) using the High-capacity cDNA Reverse Transcriptase Kit (Applied Biosystems^TM^ through Fisher Scientific, Loughborough, UK. The primer pairs were designed appropriately to the Minimum Information for Publication of Quantitative Real-Time PCR Experiments (MIQE) guidelines [18] (*Supplemental Table 1). HPRT1, MMP2 and TNFSF11* were designed by Bakkalci et al [10]. The primer conditions were selected appropriately with 60 °C of the annealing temperature. The primer pairs were obtained from Eurofins Genomics (Ebersberg, Germany). The target gene was amplified using the iTaq^TM^ Universal SYBR® Green Supermix with a 10 µl reactions composed of 20 ng sample and 0.2 µM primer concentration. The reaction was performed on the CFX96^TM^ Touch System (both from Bio-Rad, Watford, UK) for 40 cycles. The relative gene expression normalised to the reference gene, *hypoxanthine*–*guanine phosphoribosyltransferase (HPRT1)* was analysed using the ΔCT and 2^-ΔΔCT^ method [19].

### Immunofluorescence (IF)

3D samples were fixed in 10 % neutrally buffered formalin (Genta Medical, York, UK) for 30 min and then permeabilised and blocked by 2 % Triton-X 100 and 1 % bovine serum albumin (BSA) (Sigma-Aldrich, Dorset, UK) at room temperature. BSA diluted antibodies anti-RANKL (ab45039, Abcam, Cambridge, UK) or anti-osteonectin (SPARC) (ab225716) or anti-pan cytokeratin (GTX29377, GeneTex, USA) or anti- alpha-α-SMA (sc-32251, Santa Cruz Biotechnology) were applied overnight. The secondary antibodies anti-mouse Alexa Fluor^TM^ 488 IgG H&L ab150113 or anti-rabbit Dylight® 594 ab96885, Abcam, Cambridge, UK) were added to samples for 2.5 h at room temperature. All samples were all stained for Alexa Fluor^TM^ 568 Phalloidin and counterstained with NucBlue^TM^ (Invitrogen^TM^ through Fisher Scientific, Loughborough, UK).

### Histology and Immunohistochemistry (IHC)

3D samples were fixed in formalin and processed overnight in a processor (Thermo Fisher Scientific, Loughborough, UK). The processed samples were wax embedded and sectioned into 5 µm sections, which were then baked at 64 °C for 2 h. Due to visibility of distinct tumour mass and stroma of the 3D tumouroid models (Fig. 1C), areas covering tumour mass and stroma boundary were sectioned. Haematoxylin and eosin (H&E) staining was conducted after the deparaffinisation of the samples. The slides were covered by the mounting medium for imaging. IHC staining was conducted for the BRAF V600E antibody (ab2284, Abcam) and rabbit anti-human RANKL polyclonal antibody (ab9957; Abcam; 1:50) and a Tris-based buffer, CC1 pre-treatment. The Ventana Benchmark Ultrainstrument, using the Ventana OPtiview DAB detection Kit (760–124) was used for counterstaining and colour development.

### Immunohistochemistry analyses of patient samples

Immunohistochemistry (IHC) was performed on ameloblastoma cases (n = 21, ethical approval 07/H1309/150) as previously described [20]. Sections were deparaffinised in xylene (10 min), rehydrated in 100 % ethanol (10 min), incubated for endogenous peroxidase blocking with 3 % hydrogen peroxide in methanol (20 min) and followed by a PBS wash (5 min). Heat-induced antigen retrieval was carried out using 10 mM sodium citrate buffer (pH 6) by microwaving for 5 min and cooled for 3 min to prevent bone detachment. Post PBS wash, tissue was blocked with 100 % goat serum at room temperature (RT) for 30 min. Tissue sections were incubated with rabbit anti-human RANKL polyclonal antibody (ab9957; Abcam; 1:50) diluted in 100 % goat serum at 4 °C in a humidified chamber overnight. Unbound primary antibody was washed off prior to secondary biotinylated antibody (Rabbit IgG) incubation for 30 min at RT in accordance with the manufacturer’s specifications (Vectastain Elite ABC-HRP Kit; PK-6101; Vector Laboratories, Burlingame, CA, USA). ABC solution was applied for 30 min at RT and the slides were washed in PBS prior to chromogen staining with DAB (SK-4100; Vector Laboratories). Sections were counterstained with Harris’ haematoxylin (Thermo Electron Corporation, Loughborough, UK) and mounted in DPX (06522; Merck, Dorset, UK).

Whole slide images (WSI) were acquired using a Leica Aperio CS2 Scanner (Leica Biosystems, Milton Keynes, UK). Staining was quantified using QuPath software [21], where five random regions of interest were selected (area 0.15 mm^2^). The stromal area adjacent to the tumour was also analysed. The stromal regions were determined as the areas with no epithelial cells. In patient tissue, there is clear distinction between tumour and its stroma. In 3D models, the boundary that separates the tumour mass and its surrounding stroma was highlighted.

### Enzyme-Linked immunoabsorbent assay (ELISA)

RANKL protein was quantified from the cell culture supernatant by using Human TNFSF11 ELISA Kit (RANKL) (ab213841, Abcam, Cambridge, UK). Media samples were collected in triplicates per condition and stored in −80 °C. The manufacturer’s protocol was followed throughout, and plate analysis was performed on the TECAN M200 PRO Microplate Reader (Männedorf, Switzerland).

### Statistical analysis

GraphPad Software (La Jolla, CA, USA) was used for statistical analyses with a minimum of three experimental repeats. Normality test was completed using Shapiro-Wilk test (n ≥ 3) or D’Agostino test (n ≥ 8) for all data sets. Based on the normality test, appropriate statistical significance tests were chosen (details have been provided in the figure legends). The data in the graphs were presented as mean ± standard error mean (SEM) and in the text as mean ± standard deviation (SD). Statistical significance was determined as p-value <0.05.

## Results

### Adding stromal cells to ameloblastoma tumouroids change invasion and resorption profile of ameloblastoma cells

To determine whether the presence of a stromal compartment with cells is important, cellular (fibroblast and osteoblast) and acellular compartments were engineered in complex multi-compartment tumouroids. Ameloblastoma tumour masses, composed of either AM-1 and AM-3 cells were cast in between different stromal compartments, including acellular, lung fibroblasts (MRC5), gingival fibroblasts (HGF) stroma or human osteoblasts (hOB). Phalloidin staining was used to differentiate between tumour and stromal cells. Since there was no stromal cells in the 3D tumouroids with acellular stroma, no phalloidin staining was detected (Fig. 2A-D).

Initiallytwo different fibroblast types were used to assess the significance of having relevant stromal cells. Both AM-1 and AM-3 cells invaded greater distances in HGF stroma compared to non-relevant MRC5 stroma by day 14 (Fig. 2E & F). In particular, the invasion of AM-3 cells was ∼6.5-fold higher in HGF stroma compared to MRC5 stroma (p < 0.00005) (Fig. 2F). AM-3 cells preserved their morphology in both MRC5 and HGF stroma (Fig. 2A & B).

To further validate this, ameloblastoma tumour masses cultured alongside different stroma were tested for the expression of RANKL *(TNFSF11)* which is the main mechanism identified for ameloblastoma-induced bone resorption (Fig. 2D). No significant difference in *TNFSF11* was observed in MRC5 stroma compared to acellular stroma*.* Whereas *TNFSF11* expression by both AM-1 and AM-3 cells was significantly upregulated in HGF stroma (p < 0.00005) (Fig. 2G & H). Based on the preliminary invasion and gene expression results of MRC5 stroma, HGFs were chosen as an appropriate fibroblast stroma for the purpose of this study.

When HGFs were cultured in 3D, they presented an elongated morphology and stress fibres over time in 3D (*Supplemental* Fig. 1). The next step was to assess the invasion of ameloblastoma cells from the tumour mass into the surrounding stroma composed of either acellular, HGFs or hOBs (Fig. 2A-D). AM-1 cells invaded significantly longer distances in HGF stroma (349 ± 38 μm)compared to hOB stroma (183 ± 38 μm) (p < 0.05) (Fig. 2E). The invasion distance of AM-3 cells in HGF stroma was significantly greater than invasion in acellular stroma (303 ± 91 μm) (p < 0.05). The invasion of AM-3 cells in hOB stroma was ∼ 2-fold greater than AM-1 cells in hOB stroma (p < 0.005) (Fig. 2 E&F). Thus, a relevant cellular stromal compartment enhanced ameloblastoma invasion.

**Fig. 3:**
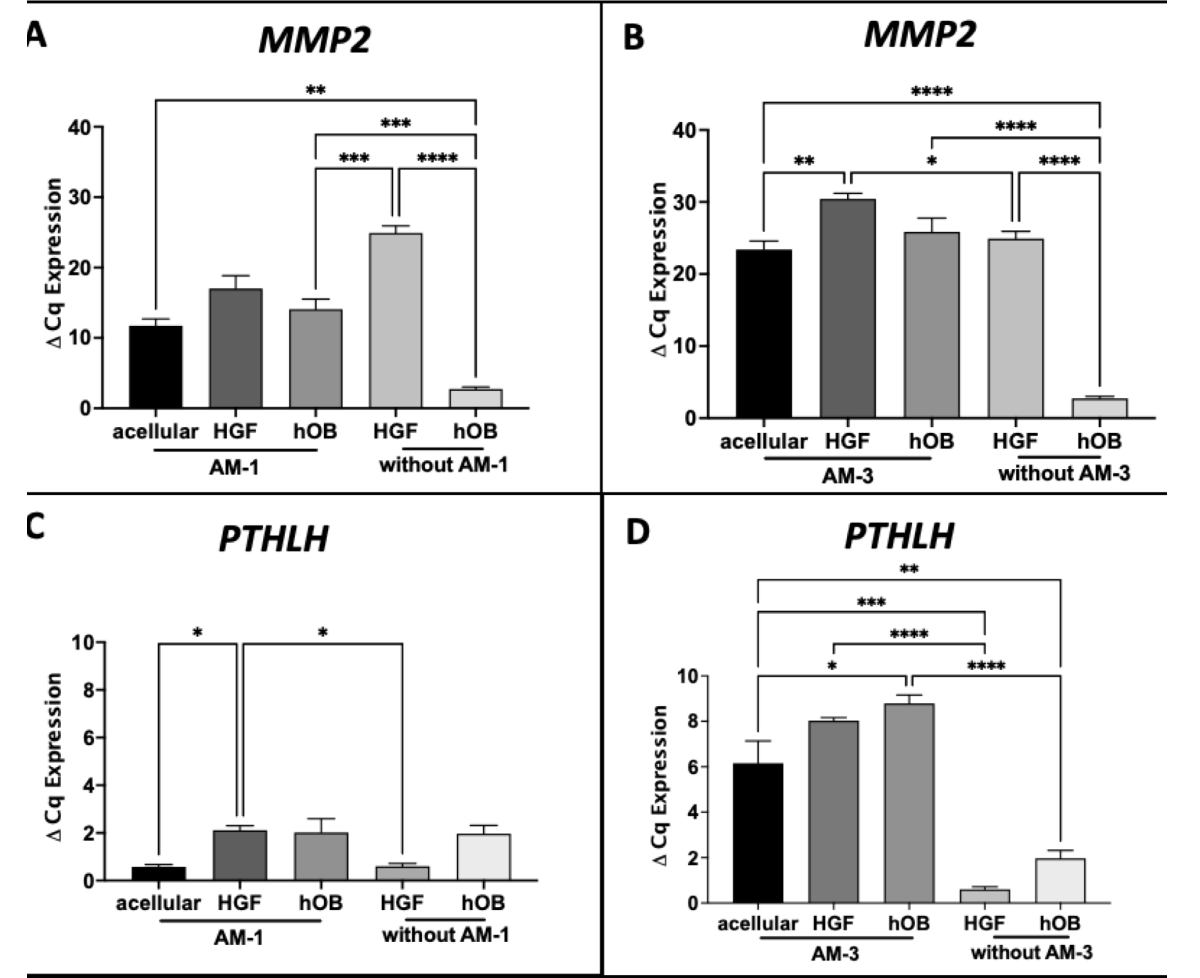
The mRNA expression of (A&B) MMP2 and (C&D) PTHLH in AM-1 and AM-3 tumoroids with either acellular stroma, HGF, or hOB stroma and the controls without AM cells. The controls are 3D cultures of either HGF or hOB by themselves at day 14. N = 3. One-Way ANOVA, Dunnet’s Post Hoc; p-values 0.05 < *, 0.005 < **, 0.0005 < *** and 0.00005 < ****.

The effects of different stroma types on the expression of invasion and bone resorption markers were also investigated. HGFs did not express *TNFSF11* gene and hOB expressed higher levels of the *TNFSF11* gene in 3D (p < 0.005). *TNFSF11* expression was upregulated in AM-1 tumouroids with HGF (p < 0.05) and hOB stroma (p < 0.0005) compared to acellular stroma (Fig. 2 *G*). *TNFSF11* expression was upregulated by ∼ 3-fold in AM-3 tumouroids with HGF stroma (p < 0.005) and ∼ 4-fold with hOB stroma (p < 0.0005) compared to AM-3 tumouroids with acellular stroma *(*Fig. 3 *H).* To measure RANKL released in culture medium, RANKL ELISA was performed on day 14 culture supernatant and no statistically significant differences in RANKL protein levels were seem among different stroma AM-1 tumouroids with different stroma. However, AM-3 cells showed altered RANKL levels. RANKL levels were ∼2-fold higher in AM-3 tumouroids with HGF stroma (p < 0.05) and 3-fold higher in AM-3 tumouroids with hOB stroma (p < 0.005) compared to AM-3 tumouroids with acellular stroma. HGFs did not release any RANKL on their own in 3D compared to hOBs, which released 150.3 ± 49.60 pg/mL in 3D (Fig. 2I).

*MMP2* expression was used to validate the observed invasion of AM cells into their surrounding stroma. When each cell type was cultured individually in 3D, HGFs expressed higher *MMP2* than hOBs and AM-1 cells (P < 0.0005)*.* AM-3 tumouroids with all stroma types expressed higher levels of *MMP2* compared to AM-1 tumouroids. Different stroma types did not cause any statistically significant difference in *MMP2* expression for AM-1 cells (Fig. 3A). AM-3 cell expressed higher *MMP2* in HGF stroma compared to acellular stroma (p < 0.005) (Fig. 3B). One of the bone resorption driver gene, Parathyroid Hormone Like Hormone *(PTHLH)* expression was higher in AM-3 tumouroids compared to AM-1 tumouroids (p < 0.005) in all stroma types. HGF stroma caused upregulation of *PTHLH* in AM-1 cells (P < 0.05) and hOB stroma induced upregulation of *PTHLH* in AM-3 cells (P < 0.05) (Fig. 3 K & L).

### Ameloblastoma histopathological patterns can be mimicked in 3D biomimetic tumouroid models

**Fig. 4:**
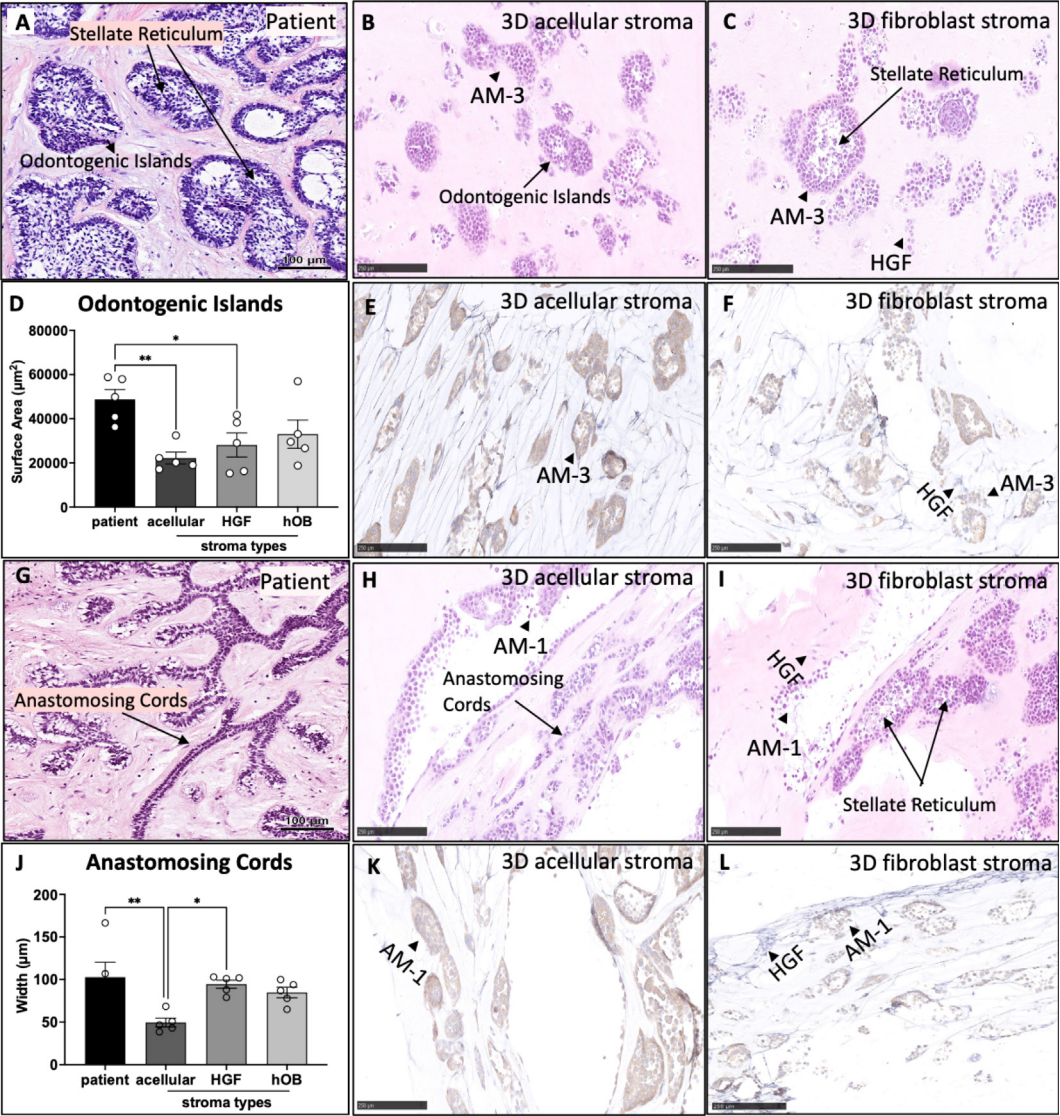
Comparison of AM tumoroid models with patient samples. H&E of (A) patient with follicular ameloblastoma scale bar = 100 µm, (B) acellular (C) HGF stroma AM-3 tumouorids, scale bar = 250 µm. (D) The surface area of the odontogenic islands formed in patients and in 3D AM-2 tumouroids with acellular and HGF stroma. BRAF IHC of (E) acellular and (F) HGF stroma of AM-3 tumouroids, scale bar = 250 µm. H&E of (G) patient with plexiform ameloblastoma, scale bar = 100 µm, (H) acellular AM-1 tumouroid, (I) AM-1 tumouroid with HGF stroma at day 14, (J) The width of the anastomosing cords formed in patients and in 3D AM-1 tumouroids with acellular and HGF scale bar = 250 µm. BRAF IHC of (K) acellular and (L) HGF stroma of AM-3 tumouroids scale bar = 250 µm. N = 3. One-Way ANOVA, Dunnet’s Post Hoc; p-values 0.05 < *, 0.005 < **, 0.0005 < *** and 0.00005 < ****.

Histology analysis of patient tissue with the follicular histopathological phenotype showed the formation of variably sized epithelial odontogenic islands with peripheral palisading and central stellate reticulum like areas (Fig. 4A). Similar islands were also observed in all AM-3 tumouroids regardless of their stroma type (Fig. 4B & C). IHC staining for *BRAF V600E* of AM-3 tumouroids showed that AM-3 cells were *BRAF* positive and HGFs were *BRAF* negative (Fig. 4E & F). HGFs aligned around the AM-3 odontogenic epithelial islands (Fig. 4F). The surface areas of the islands were compared from the H&E sections of the tumouroid models and patient samples. The island sizes in patients were significantly different ∼2.2-fold bigger than in AM-3 tumouroid with acellular stroma and ∼1.7-fold than AM-3 tumouroid with HGF stroma (p < 0.05). AM-3 cells appeared to form bigger odontogenic epithelial islands in HGF stroma and hOB stroma compared to acellular stroma, however this result was not statistically significant (Fig. 4D).

Histological sections from patients with plexiform histological subtype displayed formation of anastomosing cords and strands, whereas epithelial cells were aligned and formed branch-like structures (Fig. 4G). A similar pattern was consistently seen in all AM-1 tumouroid models (Fig. 4H & I & K). The HGFs showed parallel alignment to AM-1 cords (Fig. 4L). The widths of the cords were evaluated, and AM-1 cells formed thicker cords in HGF stroma than acellular stroma (p < 0.05) and in hOB than acellular stroma. The width anastomosing cords in patients were larger than AM-1 tumouroids with acellular stroma (p < 0.05) (Fig. 4J).

### Immunohistochemistry analysis shows RANKL expression in patient ameloblastoma tumour and stroma

**Fig. 5:**
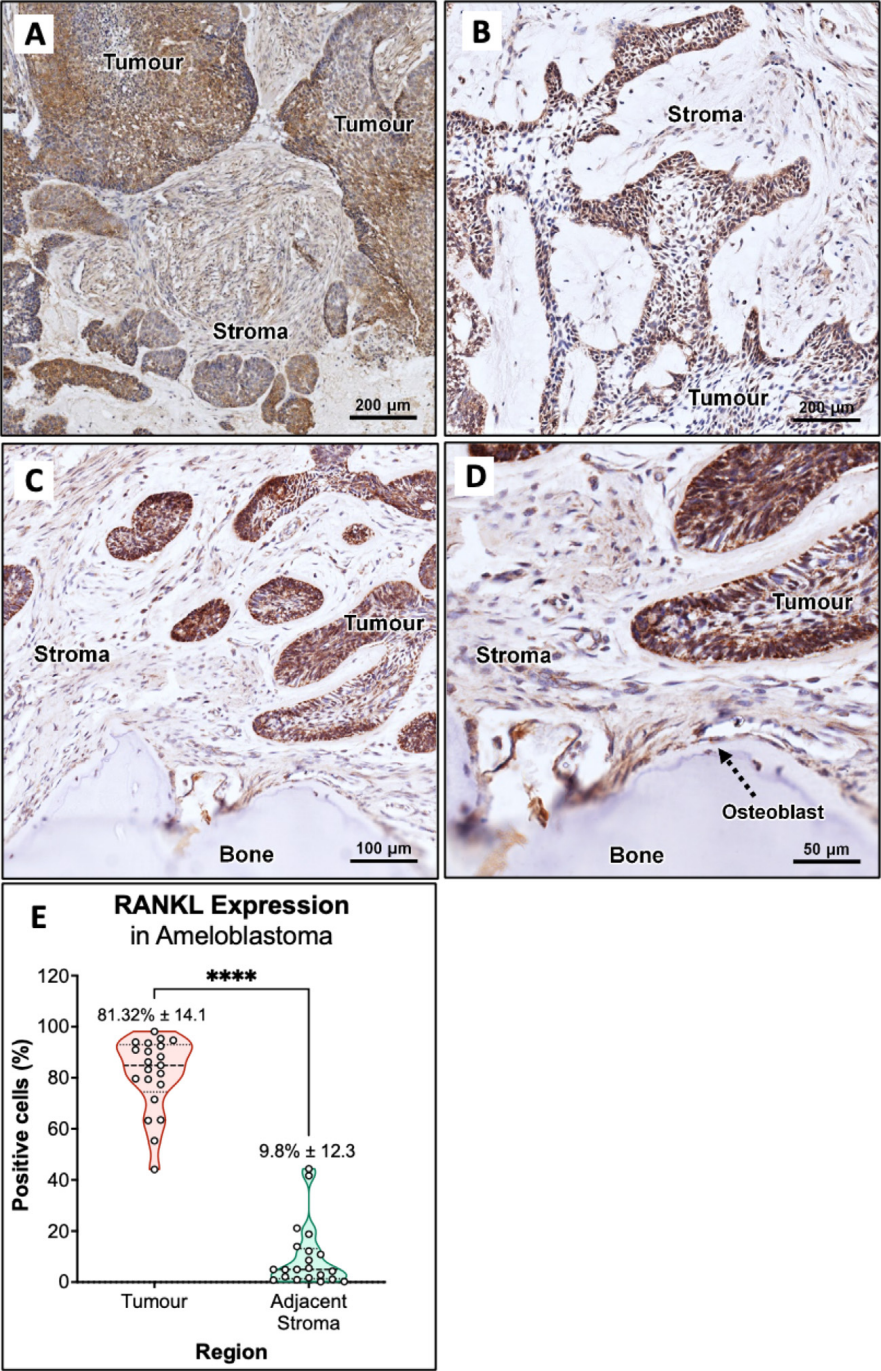
RANKL expression in ameloblastoma. Representative photomicrographs showing RANKL immunohistochemical staining in tumour and stroma of ameloblastoma FFPE tissue sections at (A&B) 100×, (C) 200× and (D) 400× total magnifications. Scale Bars for (A&B) = 100 µm, for (C) = 50 µm and for (D) = 20 µm. (E) Violin plot of RANKL-positive cell detection shows greater percentage in the tumour region compared to the adjacent stroma in the ameloblastoma. N = 3 with 5 random regions of interest were selected.

**Fig. 6:**
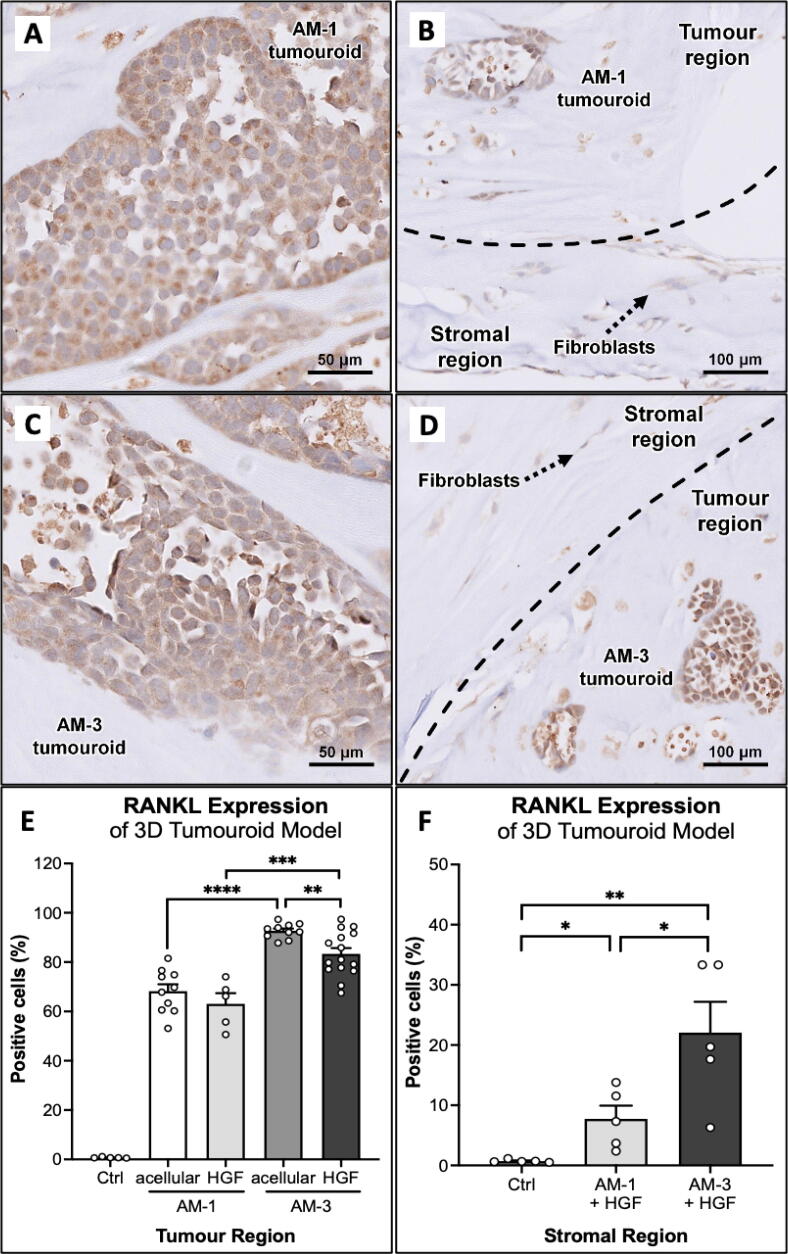
RANKL expression of the ameloblastoma 3D tumouroid models, where (A) AM-1 tumouroid with acellular stroma (total magnification 400×), (B) AM-1 tumoroid with HGF stroma (total magnification 200×), (C) AM-3 tumouroid with acellular stroma (total magnification 400×), and (D) AM-3 tumoroid with HGF stroma (total magnification 200×). RANKL expression quantification for the (E) tumour and (F) stromal region by using the QuPath software. A significant expression can be observed between the ameloblastoma cell lines (AM-1 vs AM-3) and type of stroma (acellular vs HGF stroma), including stromal expression. Data presented with mean ± SEM, p-values 0.05 < *, 0.005 < **, 0.0005 < ***. N = 3 with 5 random regions of interest were selected.

To explore the association between ameloblastoma and the bone resorption marker RANKL, IHC for RANKL was performed on 21 ameloblastoma cases. RANKL staining was seen in all selected cases with varying degrees of expression (Fig. 5A&B). The quantifications of the positive cells detected in the tumour and stroma are presented in *Supplemental Table 2* and *Supplemental Fig. 2*. Based on the positive cell quantification analysis, high expression was observed in the tumour (81.31 % ± 14.1) with medium to low expression in the stroma (9.8 % ± 12.3) (Fig. 5 E). With staining largely seen fibroblast surrounding the tumour islands (Fig. 6C & D).

### Compartmentalised 3D tumouroid model recapitulates the tumour microenvironment

RANKL expression was further assessed in AM tumouroids with acellular and HGF stroma using IHC. All AM-1 and AM-3 tumouroids with acellular and HGF stroma positively stained for RANKL compared to the control with the tumour islands showing a higher percentage of positive cells compared to the stroma (Fig. 6), *(Supplemental Fig. 3).* In AM tumouroids with HGF stroma, fibroblasts adjacent to the tumour region of the 3D tumouroid showed weak RANKL expression (Fig. 6B & D), *(Supplemental Table 3 &4).* In acellular stroma, AM-3 (92.67 % ±3.02) showed a significantly higher percentage of RANKL-positive cells compared to AM-1 (68.19 % ± 8.82) (p < 0.0005) (Fig. 6E). A similar pattern was observed in 3D tumouroid with HGF stroma, where AM-1 (63.08 % ± 9.65) had a lower percentage of RANKL-expressing cells compared to AM-3 (83.35 % ± 9.05) (p < 0.0005) (Fig. 6E). For 3D tumouroid using AM-3 cells, the acellular stroma showed a higher percentage of RANKL- positive cells compared to the tumouroid with HGF stroma (p < 0.005). Stromal analysis of the 3D tumouroid shows a higher number of RANKL-positive fibroblasts in the AM-3 (22.06 % ± 11.48; p < 0.05) (Fig. 6F).

## Discussion

The TME plays a key role in tumour progression and response to treatment and therefore needs to be mimicked *in vitro* to develop 3D biomimetic tumour-stroma models. This study focused on using different stromal cells to simulate the complexity of the TME in 3D ameloblastoma tumour models. To achieve this native ameloblastoma stromal cell populations, fibroblasts, and osteoblasts, were used in the stromal compartment of the tumouroid models. The addition of primary osteoblasts into dense collagen results in mineralisation and bone formation corresponding to an increase in the stiffness of collagen which is also a hallmark associated with tumour growth [22].

The initial step of this work was to assess whether using different fibroblast populations would induce different changes in ameloblastoma behaviour within the tumouroids. One of the significant findings of this work was that only native fibroblast stroma (i.e. human gingival fibroblasts/HGFs), enhanced ameloblastoma invasion whereas non-native stroma (comprising lung fibroblasts MRC5 cells) failed to do so. Furthermore, MRC5 cells also did not induce any significant change in gene expression of a bone resorption marker, RANKL *(TNFSF11)* (Fig. 2E-H). Our data with the HGF stroma is consistent with previously published work on how stromal cells promote ameloblastoma invasion in other 3D models [12]. Fuchigami et al. 2018 reported histological similarity in H&E images of follicular ameloblastoma samples and microscopic analysis of the immunofluorescence images of 3D AM-3 and fibroblast hemispheres [12]. The unique feature of our model is that it allows direct comparison to patient samples using histology techniques, as the model can be fixed and sectioned just like tissue samples. H&E and IHC staining of the 3D tumouroids showed alignment of HGFs around the ameloblastoma cells. This alignment led to the formation of complex, layered odontogenic islands and thick anastomosing cords as seen in patient tissue (Fig. 4).

The second main finding of this study was that the addition of the native stromal cells; HGFs and hOBs significantly increased ameloblastoma invasion and the expression of bone resorption markers (Fig. 2). *MMP2* expression in all ameloblastoma tumouroids supported invasiveness of ameloblastoma cells. Higher expression of *MMP2* in HGFs indicated that HGFs might have matrix remodelling properties. A recently published study has reported that the active ECM remodelling by ameloblastoma cells induces tumour invasion. This active remodelling was thought to involve myofibroblasts in ameloblastoma stroma [23]. The matrix remodelling properties of HGFs may be the reason of greater invasion of ameloblastoma cells in our 3D tumouroids. Upregulation of a bone remodelling regulatory gene, *PTHLH* in 3D tumouroids with fibroblast and osteoblast stroma compared to acellular stroma supported the hypothesis of increase in biomimicry with stromal complexity.

RANKL has been a popular marker to study ameloblastoma-induced bone resorption due to its abundance in ameloblastoma tumour [8], [9], [24]. This study shows that *TNFSF11 (RANKL)* expression was significantly upregulated in ameloblastoma tumouroids and further supported by the RANKL ELISA results used to measure soluble RANKL in the culture supernatant (Fig. 2). For better localisation of RANKL expression, the 3D tumouroids and patient samples were stained for RANKL using which showed higher RANKL expression in tumour cells compared to stromal cells (p < 0.0001). The same expression profile was observed in 3D ameloblastoma tumouroids with HGF stroma. HGF stroma did not seem to impact on RANKL expression in the tumour region compared to acellular stroma. This might be due to higher soluble RANKL production in ameloblastoma tumouroids with HGF stroma.

In conclusion, this novel study shows that stromal cells play a key role in developing biomimetic 3D models of AM. RANKL expression patterns that are seen in patient samples, which are believed to be instrumental to the ability of AM cells to cause bone invasion and resorption, can be mimicked in stroma cell-containing 3D tumouroids but not in 2D models. Future work will focus on utilising different stroma models for testing potential therapeutics and combining different stromal cell populations in the same model.

## Funding

D.B. receives funding from BISS Charitable Foundation.

## Declaration of Competing Interest

The authors declare that they have no known competing financial interests or personal relationships that could have appeared to influence the work reported in this paper.

## Data Availability

Data will be made available on request.
