# Supplementary material for: Modelling stromal compartments to recapitulate the ameloblastoma tumour microenvironment
Source: Matrix Biol Plus. 2022 Nov 21;16:100125. doi: 10.1016/j.mbplus.2022.100125 (PMC9703037; doi:10.1016/j.mbplus.2022.100125)
Supplement: Supplementary data 1 [file mmc1.docx]

| *Gene*  *Primer Pair* | Product Size (bp) | Number of Standards | Efficiency |
| --- | --- | --- | --- |
| *HPRT1 (Hypoxanthine-guanine phosphoribosyltransferase)*  *F' CCTGGCGTCGTGATTAGTGATG*  *R' TGAGCACACAGAGGGCTACAATG* | 190 | 4 | 90.20% |
| *MMP2 (Matrix Metalloproteinase 2)*  *F' TCCAAGTCTGGAGCGATGTGAC*  *R' TGAGCCAGGAGTCCGTCCTTAC* | 136 | 3 | 101.40% |
| *TNFSF11 (TNF Superfamily Member 11)*  *F' CGCCAGCAGAGACTACACCAAG*  *R' TGCGCTCTGAAATAGAAGAACAGG* | 248 | 5 | 101.70% |
| *PTHLH (Parathyroid hormone like hormone)*  *F' TCTGAACATCAGCTCCTCCATGAC*  *R' CTTTGTGTTGGGAGAGGGCTTG* | 153 | 5 | 101.70% |

Supplemental Table 1: Primer pairs designed for AM cells and their efficiency test results with the number of standards and the percentage of efficiency.

*
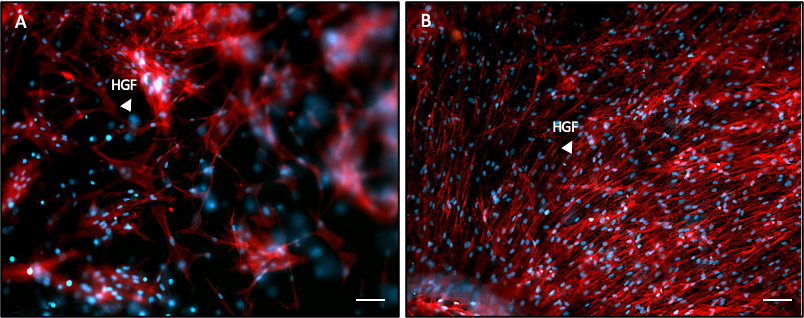
*

*Supplemental Figure 1: Human Gingival Fibroblasts in 3D. Images captured at (A) day 7 and at (B) day 14. Red = Phalloidin, blue = DAPI, scale bars = 50 µm and 100 µm respectively.*

**Osteoblast**

|  | Average positive cells (%) | | Average Number of Detection per ROI | |
| --- | --- | --- | --- | --- |
| **No** | **Tumour** | **Stroma** | **Tumour** | **Stroma** |
| 1 | 90.98 | 8.58 | 1444 | 968 |
| 2 | 90.30 | 2.83 | 1114 | 164 |
| 3 | 86.19 | 4.95 | 1563 | 566 |
| 4 | 88.23 | 21.09 | 1492 | 819 |
| 5 | 93.96 | 18.75 | 1863 | 1222 |
| 6 | 71.48 | 4.95 | 1609 | 228 |
| 7 | 95.32 | 41.70 | 1619 | 496 |
| 8 | 44.12 | 0.80 | 961 | 822 |
| 9 | 63.48 | 10.85 | 866 | 727 |
| 10 | 79.36 | 2.09 | 997 | 363 |
| 11 | 77.39 | 5.49 | 1210 | 348 |
| 12 | 81.65 | 0.25 | 1462 | 315 |
| 13 | 84.86 | 1.63 | 1160 | 398 |
| 14 | 55.33 | 0.17 | 1497 | 483 |
| 15 | 98.14 | 13.97 | 1569 | 696 |
| 16 | 79.66 | 4.28 | 1257 | 450 |
| 17 | 92.43 | 44.38 | 1590 | 508 |
| 18 | 63.30 | 0.94 | 1141 | 788 |
| 19 | 93.61 | 1.11 | 1788 | 393 |
| 20 | 83.24 | 12.17 | 932 | 288 |
| 21 | 94.66 | 4.92 | 1172 | 899 |
| **AVERAGE** | **81.32** | **9.80** | **1348** | **569** |
| **SD** | **14.10** | **12.27** | **284** | **269** |

*Supplemental Table 2: Average percentage of positive cells per selected region of interest (ROI) in each case*

*Supplemental Figure 2: Heatmap of the average positive cell detection for each case.*

| Tumour region of the 3D tumouroid model | | | | | |
| --- | --- | --- | --- | --- | --- |
|  |  | AM-1 | | AM-3 | |
|  | Ctrl | AS | HGF | AS | HGF |
|  | 1.16 | 76.57 | 74.07 | 94.94 | 85.83 |
|  | 0.70 | 63.37 | 50.63 | 93.95 | 70.49 |
|  | 0.63 | 60.57 | 66.18 | 93.52 | 77.78 |
|  | 0.68 | 74.84 | 68.82 | 92.24 | 79.80 |
|  | 0.52 | 60.24 | 55.71 | 92.10 | 79.11 |
|  |  | 53.14 |  | 95.56 | 94.44 |
|  |  | 69.75 |  | 97.30 | 97.36 |
|  |  | 67.81 |  | 90.66 | 91.67 |
|  |  | 74.07 |  | 88.65 | 89.96 |
|  |  | 81.54 |  | 87.78 | 94.16 |
|  |  |  |  |  | 80.99 |
|  |  |  |  |  | 77.08 |
|  |  |  |  |  | 87.71 |
|  |  |  |  |  | 67.48 |
|  |  |  |  |  | 76.44 |
| **AVERAGE** | **0.74** | **68.19** | **63.08** | **92.67** | **83.35** |
| **STDEV** | **0.24** | **8.82** | **9.65** | **3.02** | **9.05** |
|  |  |  |  |  |  |
|  |  |  |  |  |  |
|  | Ctrl | AM-1 AS | AM-1 HGF | AM-3 AS | AM-3 HGF |
| Ctrl |  | 0.000000 | 0.000001 | 0.000000 | 0.000000 |
| AM-1 AS |  |  | 0.323479 | 0.000000 | 0.000392 |
| AM-1 HGF |  |  |  | 0.000001 | 0.000457 |
| AM-3 AS |  |  |  |  | 0.004771 |
| AM-3 HGF |  |  |  |  |  |

*Supplemental Table 3:* *Percentage of positive cells per selected region of interest of the 3D tumouroid model tumour region with statistical analysis (green box = statistically significant).*

| Stromal region of the 3D tumouroid model | | | |
| --- | --- | --- | --- |
|  |  | HGF | |
|  | Ctrl | AM-1 | AM-3 |
|  | 1.156 | 11.54 | 17.65 |
|  | 0.7042 | 3.704 | 33.33 |
|  | 0.6289 | 7.317 | 33.33 |
|  | 0.6757 | 2.381 | 6.293 |
|  | 0.5181 | 13.79 | 19.7063 |
| **AVERAGE** | **0.74** | **7.75** | **22.06** |
| **STDEV** | **0.24** | **4.90** | **11.48** |
|  |  |  |  |
|  |  |  |  |
|  | Ctrl | AM-1 | AM-3 |
| Ctrl |  | 0.01277 | 0.0032045 |
| AM-1 |  |  | 0.0334797 |
| AM-3 |  |  |  |

*Supplemental Table 4: Percentage of positive cells per selected region of interest of the 3D tumouroid model stromal region with statistical analysis (green box = statistically significant).*


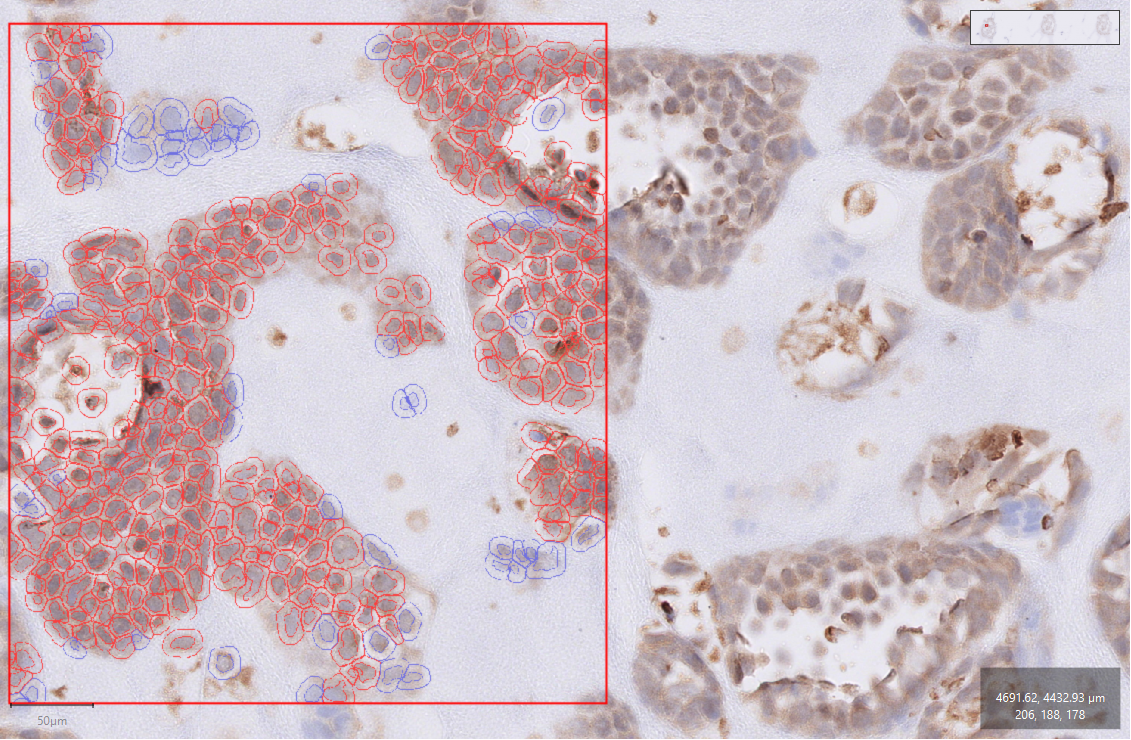

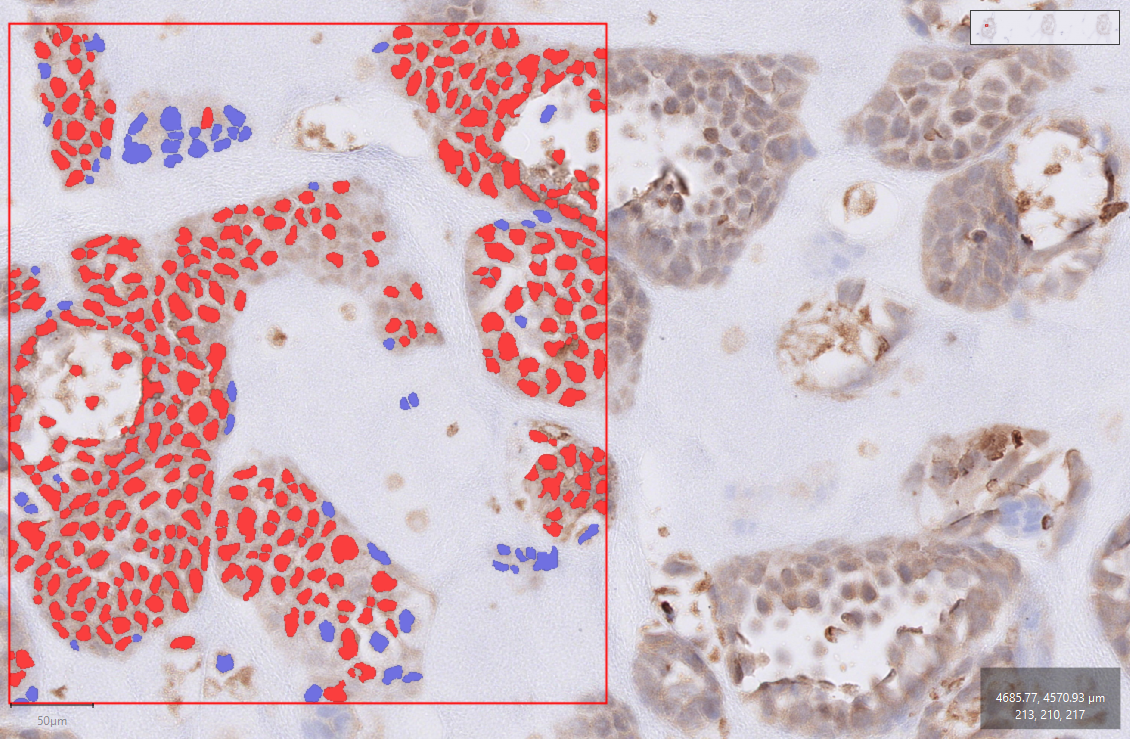


*Supplemental Figure 3: Representative image of positive cell detection for RANKL expression in the 3D tumouroid model by using the QuPath software. Mean DAB staining int the cell compartment is considered a positive staining. Red = positive RANKL expression while blue = no expression.*
